# Mental Health in Childhood and Adolescence: The Role of Polyunsaturated Fatty Acids

**DOI:** 10.3390/biomedicines9080850

**Published:** 2021-07-21

**Authors:** Paola Bozzatello, Cecilia Blua, Paola Rocca, Silvio Bellino

**Affiliations:** Department of Neuroscience, University of Turin, 10126 Turin, Italy; paola.bozzatello@unito.it (P.B.); cecilia.blua@unito.it (C.B.); paola.rocca@unito.it (P.R.)

**Keywords:** polyunsaturated fatty acids, psychiatric disorders, childhood, adolescence, schizophrenia, mood disorders, anxiety disorders, attention deficit hyperactivity disorder, substance use disorder, borderline personality disorder

## Abstract

There is increasing awareness of the importance of polyunsaturated fatty acids (PUFAs) for optimal brain development and function. In recent decades, researchers have confirmed the central role of PUFAs in a variety of patho-physiological processes. These agents modulate the mechanisms of brain cell signalling including the dopaminergic and serotonergic pathways. Therefore, nutritional insufficiencies of PUFAs may have adverse effects on brain development and developmental outcomes. The role of n-3 PUFAs has been studied in several psychiatric disorders in adulthood: schizophrenia, major depression, bipolar disorder, anxiety disorders, obsessive-compulsive disorder, post-traumatic stress disorder, attention deficit hyperactivity disorder (ADHD), autism spectrum disorders, eating disorders, substance use disorder, and borderline personality disorder. In contrast to the great number of studies conducted in adults, there are only limited data on the effects of n-3 PUFA supplementation in children and adolescents who suffer from mental disorders or show a high risk of developing psychiatric disorders. The aim of this review is to provide a complete and updated account of the available evidence of the impact of polyunsaturated fatty acids on developmental psychopathology in children and adolescents and the effect of fatty acid supplementation during developmental milestones, particularly in high-risk populations of children with minimal but detectable signs or symptoms of mental disorders.

## 1. Introduction

In the last decade, there has been growing interest in the role of nutritional factors, particularly polyunsaturated fatty acids (PUFAs) in the development, treatment, and prevention of neurodevelopmental disorders [1,2,3,4,5,6,7].

The most important classes of PUFAs are the omega-3 fatty acids (n-3 PUFAs) and the omega-6 fatty acids (n-6 PUFAs). Among n-3-PUFAs, the forms relevant for human health are α-linolenic acid (ALA, C18:3n-3), eicosapentaenoic acid (EPA, C20:5n-3), and docosahexaenoic acid (DHA, C22:6n-3), whereas among the n-6 family, there are linoleic acid (LA, C18:2n-6), di homo-γ-linolenic acid (DGLA, 20:3n-6), and arachidonic acid (AA, C20:4n-6) [8,9]. They are essential fatty acids (EFAs) as they cannot be produced within the human body and can only be provided by diet [10]. PUFAs of both families play a significant role in the inflammatory, immune, cardiovascular, and nervous system [11,12,13,14], but derivatives of n-6 and n-3 PUFAs regulate these systems mostly in an opposing (antagonistic) manner. Generally, n-6 enhances inflammation, platelet aggregation, and vasoconstriction, whereas n-3 inhibits inflammation and platelet aggregation and enhances vasodilation [15,16]. In particular, n-3 PUFAs have been shown to play a key role as elements of phospholipids and cholesterol esters of the neuronal cell membrane, especially of dendritic and synaptic membranes, and as signalling molecules in all tissue including the brain [17]. DHA is the most prevalent polyunsaturated fatty acid (PUFA) in the central nervous system (approximatively 60% of PUFAs in neuronal membranes) [18] and is important for central nervous system development [3,19,20]. Deficiency of DHA in the early stages of life may cause changes of myelination, neurogenesis, synaptogenesis, neurotransmitter turnover, cellular differentiation and development, brain connectivity, and inflammatory reactions [18,19,20,21]. Supplementation of phospholipid precursors in animal models promotes the release of neurotransmitters such as dopamine and acetylcholine [22,23]. On the other hand, lower levels of DHA have been associated with neuronal membrane instability and dysfunctional transmission of serotonin, norepinephrine, and dopamine, which might be associated with mood and cognitive disfunction [24,25,26]. On the basis of these preclinical findings, several lines of evidence support the importance of n-3 PUFAs in the treatment of psychiatric disorders such as schizophrenia, major depression, bipolar disorder, anxiety disorders, obsessive-compulsive disorder, post-traumatic stress disorder, attention deficit hyperactivity disorder (ADHD), autism spectrum disorders, eating disorders, substance use disorder, and borderline personality disorder [27,28,29,30,31]. Furthermore, in a trans-diagnostic perspective, recent studies have pointed out that supplementation with n-3 PUFAs provides benefits on the main psychiatric symptom dimensions, particularly in domains of psychotic symptoms, affective symptoms, impulsivity, and harmful behaviours [32,33,34].

In contrast to the great number of studies conducted in adults, there are only limited data on the efficacy of n-3 PUFAs in mental disorders in children and adolescents. The aim of this review is to provide a complete and updated account of the available evidence of the impact of n-3 PUFAs on developmental psychopathology in children and adolescents, and the effect of supplementation with these fatty acids during developmental milestones, particularly in high-risk populations of children with minimal but detectable signs or symptoms of mental disorders.

## 2. Methods

**Figure 1:**
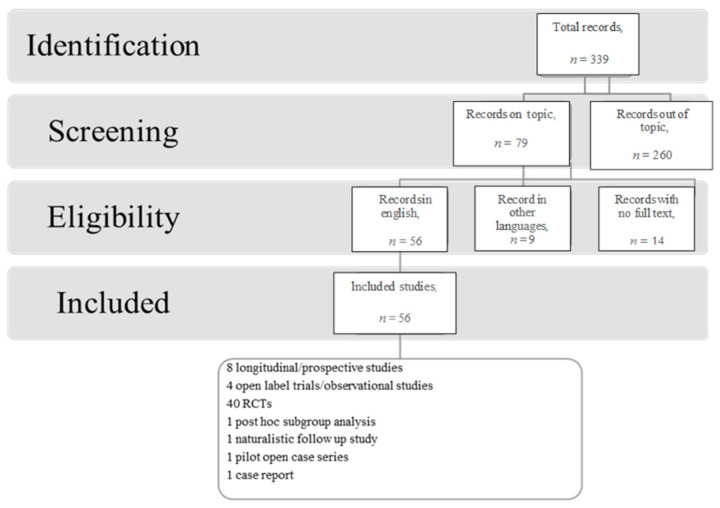
Literature search flowchart.

In March 2021, an electronic search was performed on PubMed on the role of n-3 PUFA supplementation in the treatment of psychiatric disorders in children and adolescents without any filter or MESH restriction using the following search string (“n-3 polyunsaturated fatty acids” OR “omega 3”) AND (“mental health” OR “psychiatric disorder” OR “mental disease” OR “bipolar disorder” OR “schizophrenia” OR “major depression” OR “anxiety disorders” OR “obsessive compulsive disorder” OR “post-traumatic stress disorder” OR “attention deficit hyperactivity disorder” OR “autism spectrum disorders” OR “eating disorders” OR “substance use disorder” OR “borderline personality disorder” OR “psychotic symptoms” OR “affective symptoms” OR “impulsivity” OR “harmful behaviours”) AND (“adolescent” OR “children”)). We included the following types of publications: controlled clinical trials, observational studies, longitudinal and prospective studies, cohort studies, and reviews from January 2000 until March 2021. Overlapping studies were excluded. The literature search is summarised in the flowchart (Figure 1).

## 3. Results

The search described in the previous section provided 339 records. Eligibility status for articles was determined in the following way: (1) All studies were screened on the basis of the title and abstract; and (2) papers that passed the initial screening were reviewed on the basis of a careful examination of the full manuscript content. This review included 56 records, eight longitudinal/prospective studies, four open label trials/observational studies, 40 randomised controlled trials (RCTs), one post-hoc subgroup analysis; one naturalistic follow-up study, one pilot open case series, and one case report. The review considered only articles written in English.

## 4. Discussion

### 4.1. Schizophrenia

Several studies suggest that genetic disturbances in phospholipid and prostaglandin metabolism may contribute to schizophrenia aetiology and severity [35,36]. In particular, patients with schizophrenia had lower concentrations of erythrocyte fatty acids compared to the control subjects [37] and a significant relationship between lower erythrocyte essential fatty acid concentration and greater severity of negative symptoms [38,39,40,41] were observed. Furthermore, some authors have proposed the detection of subgroups of patients with schizophrenia on the basis of the bimodal distribution of abnormalities in lipid composition [42]. In line with these assumptions, it has been argued that dysfunctional fatty acid metabolism could be involved in the aetiology of the disorder as early evidence of systemic disease before the onset of psychotic illness [38].

Over the past decades, the terms “high risk” and “ultra-high-risk” (UHR) have been used to identify adolescents and young adults who are at increased risk of developing psychotic symptoms. Among these subjects, about 22% to 40% experienced transition to psychosis within three years of initial assessment [43,44,45]. These data indicate the need for diagnostic and therapeutic interventions to prevent the onset of psychosis [46]. In this context, Cadenhead and colleagues analysed the correlation between vulnerability to metabolic abnormalities and the risk of psychosis [47]. In this study, the authors evaluated demographic information, cardiometabolic indices, prodromal symptoms, positive symptoms, negative symptoms, disorganised symptoms, and general symptoms (assessed with the Scale of Prodromal Symptoms, SOPS from the SIPS interview) and functioning in an antipsychotic free cohort at clinical high risk (CHR) for psychosis. Over 90% of the 113 CHR subjects included in the study demonstrated evidence of elevated oxidative stress. In addition, they found a relatively low (<4%) red blood cell n-3 PUFA index (% of EPA + DHA). They also found a significant association between cardiometabolic abnormalities, lower levels of n-3 PUFAs intake and increased severity of symptoms and poorer functioning, suggesting that dietary factors and systemic illness may play a role in the psychosis disease process. The association between symptom severity and decrease in red blood cell PUFAs is consistent with prior studies in chronic schizophrenia that showed similar associations with symptoms, cognitive impairment, and tardive dyskinesia [41]. Thus, treatment with n-3 PUFAs may be considered as a potential initial treatment for very young people who have a risk of developing prominent psychotic symptoms. In fact, the use of antipsychotic medication in young people at risk of psychosis is controversial [48,49], while PUFAs do not have clinically significant adverse effects. For this reason, several clinical trials [50,51,52] have been carried out to assess the efficacy of PUFA therapy in populations of young patients at risk of schizophrenia or at first episode of psychosis.

Amminger and collaborators sought to examine the effect of 12-week intervention of 1.2 g/day n-3 PUFAs or placebo in 81 individuals at ultra-high risk of psychotic disorder aged 13 to 25 years. N-3 PUFA supplementation reduced the risk of progression to psychotic disorder in young people with subthreshold psychotic states. This protective effect persisted at 12-months of follow up. In fact, only two of 41 individuals (4.9%) in the treatment group developed psychosis compared to 11 of 40 (27.5%) in the control group. The treatment group also had significantly reduced positive symptoms, negative symptoms, general psychiatric symptoms, and improved functioning compared with the placebo group [50]. Similar results were found by Pawełczyk [51] in a randomised placebo-controlled trial aimed to compare the efficacy of 26-week intervention, composed of either 2.2 g/day of n-3 PUFAs or olive oil placebo in 71 patients at first-episode schizophrenia. A fifty-percent improvement in symptom severity was achieved more frequently in the n-3 PUFA group than in the placebo group (69.4 vs. 40.0%). This finding suggests that long-term therapy with n-3 PUFAs reduced symptom severity and increased response rate, improved level of functioning and decreased the intensity of depressive symptoms [51]. Functional improvement was also confirmed in a post-hoc subgroup analysis conducted by Amminger and colleagues [53] on the same cohort of 81 UHR individuals (27 males, 54 females; mean age = 16.4, s.d. = 2.1 years) who had been previously recruited in a double-blind randomised controlled trial of n-3 PUFAs vs. thee placebo [50]. The authors demonstrated that higher levels of erythrocyte membrane ALA and more severe negative symptoms at baseline predicted functional amelioration in the treatment group. Thus, UHR patients with higher levels of α-linolenic acid may specifically benefit from PUFA supplementation. In addition, fatty acid baseline level could potentially be used to inform prognostic evaluations and treatment decisions [53]. Therefore, findings were consistent with the hypothesis that n-3 PUFA metabolism is strictly implicated in the aetiology of negative symptoms of schizophrenia [54], and with the notion that oxidative damage to lipids is connected to the process of neuroprogression and the expression of negative symptoms [55].

On the other hand, a recent multi-centre replication study did not show efficacy of n-3 PUFAs in subjects at risk of developing psychosis [52]. McGorry and colleagues conducted a double-blind, placebo-controlled, randomised clinical trial on the efficacy of 1.4 g/day n-3 PUFA supplementation for six months, followed by an additional 6-month follow-up period in 304 participants who received either n-3 PUFAs together with cognitive behavioural case management (CBCM) or the placebo with CBCM. Although n-3 PUFAs were well tolerated, they did not demonstrate an advantage over the placebo in the prevention of psychosis at 6- or 12-month follow-up evaluations. However, the transition rate of 10.5% was lower than expected. The authors stated that there are two possible explanations for this lower transition rate. First, both treatment groups received the manualised CBCM intervention and a high level of antidepressant treatment that might have produced a ceiling effect beyond which there was no scope for n-3 PUFAs to confer additional benefit. Second, the sample may have been not at a sufficiently high risk of transition.

In conclusion, early and integrated precocious treatment plans might prevent the onset and reinforcing of symptoms and dysfunction in patients with schizophrenia [56]. The investigations in this clinical population are too limited to draw any conclusions. Further studies are needed on n-3 PUFA supplementation in young people with subthreshold psychotic states in order to verify the reduction of the progression rate into psychosis.

Results of the RCTs are displayed in Table 1.

### 4.2. Bipolar Disorder

Bipolar disorder in paediatric patients is a recurrent, difficult-to-treat, mental illness characterised by a predominant mood state of irritability and often mixed, rapid-cycling, and psychotic symptoms [57]. Despite the large community interest in n-3 PUFAs as a treatment for mood disorders and the increasing attention paid to early onset of mental diseases, there are still few randomised controlled trials on the use of n-3 PUFAs in juvenile bipolar disorder.

A cross sectional study performed by McNamara and collaborators [58] investigated the role of n-3 PUFAs (EPA and DHA) in youths with or at varying risk for developing mania. They analysed erythrocyte fatty acid composition in healthy adolescents (*n*  =  28), asymptomatic adolescents at high risk (*n* = 30) and at ultra-high risk (*n* = 36) for developing mania, and first-episode adolescent bipolar manic patients (*n*  =  35). The authors observed that increasing risk for developing bipolar disorder was associated with deficits in the erythrocyte’s EPA and DHA levels and stated that low levels of n-3 PUFAs can be considered a prodromal risk biomarker for bipolar disorder in young subjects. This theory has found support from a prospective open label trial performed by Wozniak and colleagues [59] that consisted of an 8-week open-label treatment with 1290–4300 mg combined EPA (eicosapentaenoic acid) and DHA (docosahexaenoic acid) in a population of 20 children, six to 17 years of age, with a diagnosis of paediatric bipolar disorder. Youths treated with n-3 PUFAs showed an increase in these fatty acids both in plasma and in red blood cell membranes over the course of the study, indicating the capacity of the membrane to take up the n-3 PUFA supplementation. The supplementation also resulted in a moderate improvement in manic symptoms as measured by the Young Mania Rating Scale (YMRS). Unfortunately, in this study, participants remained symptomatic at the endpoint, although they showed a significant reduction in YMRS scores. Results from a more recent study performed by the same investigators [60] went in the same direction. In a 12-week, randomised, double-blind, controlled clinical trial, the authors enrolled 24 children, aged between five and 12 years old, with bipolar spectrum disorders who were treated with EPA and DHA and inositol. All study subjects were randomised to receive 3000 mg (six 500-mg capsules) of n-3 PUFAs or placebo for the duration of the study, the dose depending on body weight. The results suggest that the combined treatment of n-3 PUFAs plus inositol reduced symptoms of mania and depression in pre-puberal children with mild to moderate bipolar spectrum disorders. Similar findings were obtained in 18 participants with juvenile bipolar disorder treated with n-3 PUFA supplementation [61] They treated 18 children (12 females, mean age = 16.1 ± 0.81 years; six males, mean age = 13.0 ± 1.06 years) with a supplementation of 360 mg/day EPA and 1560 mg/day DHA as an adjunct to standard pharmacological treatment for six weeks. The investigators concluded that clinician ratings of mania and depression (especially ratings of depression) were significantly lower and global functioning was higher after supplementation. According to previous studies [62,63], they also noticed that supplementation significantly increased EPA and DHA in red blood cell membranes.

In recent years, three studies have investigated the efficacy of the association between n-3 PUFAs and psychotherapeutic interventions.

Fristad and colleagues [64] made a 12-week RCT in order to compare the combination of psychoeducational psychotherapy and 2000 mg/day of n-3 PUFAs with the placebo and active monitoring in twenty three youths aged 7–14 years with subsyndromal bipolar disorders (bipolar disorder not otherwise specified, cyclothymic disorder). Combined therapy was associated with greater improvement in depressive symptoms, but not in manic symptoms. However, all participants experienced and reported a decline in manic symptoms over the course of the study. They also reported that two planned comparisons yielded effect sizes ranging from small (n-3 PUFAs vs. placebo) to large (n-3 PUFAs + active monitoring vs. placebo + active monitoring) on the YMRS. This observation may indicate potential benefits of n-3 PUFAs for co-occurring problems such as inattention, hyperactivity, and aggressive behaviour, which are assessed by the YMRS and have been reported to improve with n-3 PUFAs in prior studies [65,66]. The same research group [67] performed a follow-up study of 2–5 years after participation in the previous randomised clinical trial (RCT) to evaluate the long-lasting effects of combined therapy. The authors found that participants, regardless of the treatment group, remained comparable to the end of the RCT regarding manic symptom severity, executive functioning, and global functioning: there were no differences in YMRS scores between those who were treated with n-3 PUFAs or participated in psychotherapy after the end of the RCT compared to those who did not. In contrast, they found that those who persisted in utilising n-3 PUFAs had lower depressive symptom severity than those who did not utilise these intervention.

The third study was a 12-week RCT aimed to assess the impact of 1870 mg/day n-3 PUFAs supplementation and psychoeducational psychotherapy, each alone and in combination, on executive functions in ninety-five youths with mood disorders. Groups receiving PUFA supplementation were associated with significant improvement in executive functions over time and many of patients also demonstrated concurrent improvements in dysphoric mood, irritability, and self-esteem [68].

Results of the RCTs are displayed in Table 2.

### 4.3. Major Depression

Adolescence and early adulthood are two phases of life in which initial depressive symptoms may arise. Evidence in the literature suggests that depression rates are rising among children and adolescents, potentially driven by alterations in environmental factors [69] One of these factors might be found in lifestyle change: dietary patterns in both affluent and developing countries now consist primarily of high-energy, nutrient poor foods, and have shifted away from traditional diets including higher intakes of plant foods and quality proteins [70]. This change has resulted in an increased consumption of n-6 PUFAs and a depletion of n-3 PUFAs [71], which leads to an imbalance of fatty acid composition in plasma and erythrocytes with a negative impact on central nervous system neuronal membranes and serotonin transport.

Available data show a relationship between low levels of EPA and DHA and depressive symptoms in adulthood [72,73,74]. Rather poor evidence has been collected about n-3 PUFA levels in the tissues of depressed adolescents. To our knowledge, six studies have been performed in order to analyse the relationships between n-3 PUFAs levels and youth depression.

In a small study made by Pottala and colleagues, DHA levels in red blood cells were inversely related to depression in a sample of 150 depressed adolescents [75]. Three randomised trials (RCTs) examined the effects of treatment with fish oil in depressed adolescent patients. The first one [76] included 20 subjects who received either the placebo or fish oil (400 mg EPA and 200 mg DHA per day) for at least one month. The authors found an improvement in depressive symptoms in the group who received fish oil. The second study included 18 adolescent patients with bipolar depression [61] who received supplements containing 360 mg per day of eicosapentaenoic acid (EPA) and 1560 mg per day of docosahexaenoic acid (DHA) for six weeks. Results showed that clinician ratings of depression were significantly lower and global functioning was significantly higher after supplementation. More recently, a similar trial was performed by Trebatická and colleagues on a larger sample (N= 60) [77] The primary goal of the study was to assess the effects of n-3 PUFA fish oil emulsion (2400 mg of total n-3 PUFAs, in particular 1000 mg EPA and 750 mg DHA, EPA:DHA ratio = 1.33) in comparison with a control oil emulsion alongside the standard treatment for depression in children and adolescents suffering from depressive disorder and/or mixed anxiety depressive disorder. The authors found significant reductions in Children’s Depression Inventory (CDI) scores in patients who completed 12 weeks of n-3 PUFA supplementation compared with the placebo. This result suggests that n-3 PUFA rich fish oil may be an adjuvant supplement to standard antidepressants for the treatment of depressive disorder.

Two RCTs investigated the efficacy of n-3 PUFAs in association with psychological interventions [68,78]. Young and collaborators investigated the benefits of the association of 2000 mg/day n-3 PUFA supplementation and Individual-Family Psychoeducational Psychotherapy (PEP), a family-focused, cognitive-behavioural therapy for youths with depression. They recruited 72 children aged between seven and 14 years with depressive disorders for a 12-week trial. Participants were randomly divided into four groups: (1) PEP + n-3 PUFAs; (2) PEP in monotherapy (with pill placebo); (3) n-3 PUFAs in monotherapy; and (4) placebo pills without active intervention. Behavioural problems were assessed with the Swanson, Nolan, and Pelham-IV (SNAP-IV) scale, which assesses attention-deficit/hyperactivity disorder symptoms, oppositional defiant disorder symptoms, and overall behavioural problems, and with the Eyberg Child Behaviour Inventory (ECBI), which includes Intensity and Problem scales for child behavioural problems. The authors observed that n-3 PUFAs yielded more positive trajectories than the placebo on the SNAP-IV Hyperactivity/Impulsivity subscale and were marginally more favourable on Total and Inattention scores. Therefore, combined PEP and n-3 PUFAs supplementation and n-3 PUFA supplementation alone may play an important role in inattentive symptoms among youths with depression [78].

Vesco et al. (2018) performed a 12-week RCT in 95 youths with mood disorders including depression, bipolar disorder-not otherwise specified, and cyclothymic disorder [68]. The authors assessed the impact of 1.87 g/day n-3 PUFA supplementation and psychoeducational psychotherapy, each alone and in combination, on executive functions in youths with mood disorders. Participants receiving n-3 PUFA supplementation were associated with significant improvement in executive functions over time. Both groups receiving n-3 PUFAs (in combination and as monotherapy) demonstrated medium or better placebo-controlled effect sizes.

Results of the RCTs are displayed in Table 3.

### 4.4. Anxiety Disorders

Anxiety disorders are the most prevalent psychiatric conditions in children and adolescents [79,80]. According to the literature, the course of an early onset anxiety disorder is often chronic and increases the risk of developing additional psychopathology in adulthood such as substance use or mood disorders [81,82]. Investigations examining the effect of n-3 PUFAs on anxiety disorders in adolescence is limited and the available data are predominantly based on subjects with anxious symptoms in the absence of a diagnosis of anxiety disorder, or in clinical populations with comorbid disorders. Studies performed on individuals without a diagnosis of anxiety disorder, but with subthreshold anxiety symptoms have suggested that PUFA administration may produce an anxiolytic benefit [83,84]. In a study that enrolled 22 healthy volunteers (ages 18 to 24 years), n-3 PUFAs reduced plasma noradrenaline (norepinephrine) levels after eight weeks of supplementation with 400 mg EPA and 300 mg DHA per day and were associated with decreased activation of the hypothalamic–pituitary–adrenal (HPA) axis [83]. Therefore, n-3 PUFA supplementation might produce a stabilisation of the HPA axis, which resulted in a reduction in anxiety levels. Consistently with these results, the study conducted by Kiecolt-Glaser and colleagues [84] reported an improvement in anxiety symptoms in a sample of 68 healthy medical students (38 men and 30 women) ranging in age from 21 to 29 years, treated for 12 weeks with 2496 mg/day n-3 PUFA supplementation. In contrast, Manos and collaborators [85] conduced a pilot RCT to test the effects of 12-weeks of n-3 PUFA supplementation (2120 mg EPA/600 mg DHA) on the comorbidity of anxiety disorder and anorexia nervosa in 24 adolescents aged 12 to 21 years. No efficacy on anxiety was observed in this sample. Robinson and collaborators [86] examined the effects of adjunctive n-3 PUFA treatment (EPA 740 mg and DHA 400 mg daily) for 16 weeks in a larger sample including 50 patients, aged 15–40 years, with early psychosis and anxiety. Implementation with n-3 PUFAs was found to be mainly useful on symptoms of depression and anxiety measured with the Brief Psychiatric Rating Scale (BPRS).

In summary, further investigations are needed to specifically evaluate the potential effect of n-3 PUFAs in anxiety disorders. Available studies in children and adolescence have enrolled small sample sizes and are underpowered.

Results of the RCTs are displayed in Table 4.

### 4.5. Obsessive Compulsive Disorder

Epidemiologic studies have stated that many children suffer from subclinical obsessive-compulsive (OC) symptoms and by late adolescence OCD has a lifetime prevalence of 2% to 3% [87]. The age of onset is earlier in boys than in girls, and has a first peak around puberty and another in early adulthood [88,89] No RCTs have been published on OCD in adolescents or children. There is only one study conducted by Fux and colleagues [90], in which 2 g/day of EPA or placebo were administered for 12 weeks in augmentation of a stable dose of serotonergic antidepressant (SSRI) to 11 patients aged between 18 and 75 years. Results were unfavourable, as the augmentation with n-3 PUFAs was not associated with significant improvements of anxious, obsessive-compulsive, and depressive symptoms compared to the placebo.

### 4.6. Attention Deficit Hyperactivity Disorder

Attention Deficit/Hyperactivity Disorder (ADHD) has an estimated prevalence of 4% to 12% of school-aged children worldwide [91]. Up to 25% of children with ADHD show one or more specific learning disabilities in math, reading, or spelling. Hyperactive children experienced behavioural problems more often than normal children, temper tantrums, learning, health, and sleep problems, but also increased thirst, eczema, asthma, and other allergies that may be correlated to essential fatty acid deficiency [92]. Since the 1980s, scientific research has investigated the role of both n-3 and n-6 PUFAs in ADHD. In one of the first studies conducted by Mitchell et al. [93], the serum levels of DHA and arachidonic acid (AA) were found to be significantly lower in hyperactive children compared to the controls (44 hyperactive subjects and 45 controls). Stevens et al. [94] found that plasma and red blood cell (RBC) levels of AA, EPA, and DHA were significantly lower in 53 ADHD patients than in the 43 controls, in the absence of difference in the dietary intakes of fatty acids between children with ADHD and healthy children. These findings are in concordance with a more recent study performed by Yonezawa [95]. Authors compared the plasma PUFAs levels of 24 patients with ADHD with the standard reference levels for healthy subjects. They found that plasma concentrations of DHA, EPA, and EPA/AA were significantly lower than the normal reference range, indicating that ADHD patients present an imbalance in PUFA levels. To probe abnormalities in membrane fatty acids and biochemical alterations in ADHD patients, Ross et al. [96] measured exhalant ethane levels, a non-invasive measure of oxidative damage to n-3 PUFAs, in 10 children (aged 10.8 ± 0.8 years) diagnosed with ADHD. They found that children with ADHD exhaled increased levels of ethane, suggesting that patients with ADHD have higher rates of oxidative breakdown of n-3 PUFAs. This biochemical abnormality may underline the previously observed fatty acid deficiencies as well as provide a rationale for the use of n-3 PUFA supplementation therapy in the treatment of ADHD.

In one study [97] aimed at evaluating the relationships between resting-state EEG activity and blood concentrations of fatty acids in 46 adolescent boys with ADHD, aged between 12 and 16 years, the investigators found a positive association of DHA levels with fast frequency activity (alpha and beta) and with performance on fluency for categories (semantic memory) and an inverse correlation of EPA levels with slow frequency activity (theta) and with delayed verbal performance.

Based on this data, it has been hypothesised that supplementation with PUFAs, particularly with n-3 PUFAs, may result in an improvement in ADHD symptoms. In 2001, Voigt et al. [98] supplemented 63 6-to-12-year-old children with ADHD with either placebo or 345 mg DHA/day for four months. DHA levels in blood increased in the group receiving supplementation, but there were no significant improvements in any measure of ADHD symptoms. However, Richardson and Puri [99] showed that supplementation with a mixture of EPA (186 mg/day), DHA (480 mg/day), gamma-linolenic acid (96 mg/day), vitamin E (60 IU/day), AA (42 mg/day), LA (864 mg/day), and thyme oil (8 mg/day) for 12 weeks in forty-one children aged 8–12 years with specific learning disabilities improved seven out of 14 symptoms of ADHD (although only three were significant) compared to none for the placebo. Stevens et al. [100] supplemented fifty children with ADHD (aged between six and 13 years) with 480 mg DHA, 80 mg EPA, 40 mg AA, and 96 mg GLA per day for four months, reporting an increase in both EPA and DHA in plasma as well as an improvement in parent-rated conduct, teacher-rated attention, and oppositional defiant behaviour. Furthermore, there was a significant correlation between increased RBC n-3 PUFAs and a decrease in disruptive behaviour. Contrasting results were published by Muller and collaborators [101], who conducted a 16-week trial with 95 children (6–12 years of age) diagnosed with ADHD according to DSM-IV criteria. Supplementation with 720 mg/day of n-3 PUFAs increased the concentrations in erythrocyte membranes and improved working memory function, but had no effect on other cognitive measures and parent- and teacher-rated behaviour in the study population. More encouraging data were collected by Harding et al. [102] in a trial assessing the effect of dietary supplements including n-3 PUFAs (180 mg EPA, 120 mg DHA, and 45 mg gamma-linolenic acid per day) in 20 children with ADHD randomly assigned to receive metilphenidate (*n* = 10) or dietary supplements (*n* = 10). Although small and non-randomized, this study suggested that dietary supplementation resulted in equivalent improvements in attention and self-control as methilphenidate. Similar findings were reported by Huss and colleagues [103] who made a large observational study in which they monitored 810 children referred for attentional and behavioural problems in the age range of five to 12 years. Patients were treated with PUFAs (500 mg/day) in combination with zinc (5 mg/day) and magnesium (80 mg/day). After 12 weeks of consumption, most subjects showed a considerable reduction in symptoms of attention deficit and hyperactivity/impulsivity. Wu and colleagues [104] randomly assigned 179 children aged 7–12 years old with lower IQs (*n* = 88) or ADHD (*n* = 91) to receive ordinary eggs (control group, *n* = 90) or eggs rich in n-3 PUFAs for three months. They reported improvements both in visual acuity and in the RBC fatty acid profile in school-age children with lower IQs or ADHD who received dietary supplementation with n-3 PUFAs. Finally, Hirayama et al. [105] examined the effect of DHA supplementation in food sources for two months on symptoms of ADHD in 40 children aged between six and 12 years. On average, children received 0.5 g DHA/day versus control foods. There was no improvement of ADHD symptoms in this study. These findings seem to suggest that a combination of PUFAs is more likely to exert a positive effect on ADHD symptoms than n-3 PUFAs alone.

Results of the RCTs are displayed in Table 5.

### 4.7. Autism Spectrum Disorders

Several studies have reported an association between low plasma levels of omega-3 fatty acids and autism spectrum disorder (ASD) in children. There is a general consensus on the significant role of PUFA metabolism in neurodevelopmental disorders and on symptom improvement derived by their supplementation [106,107,108,109].

With regard to trials exploring the effect of n-3 PUFA supplementation in ASD, eight studies have been identified. Among them, half reported some improvement in ASD symptoms [110,111,112,113], whereas the other four studies did not find any significant ameliorating effect.

Focusing on the four studies reporting a significant improvement, they all had a maximum sample size of 41.

The first one was a 6-week placebo controlled pilot trial conducted by Amminger and collaborators, who investigated the effects of 1.5 g/day of n-3 PUFA (840 mg/day EPA and 700 mg/day DHA) supplementation in 13 children aged five to 17 years with ASD accompanied by severe tantrums, aggression, or self-injurious behaviours. Authors reported an advantage of n-3 PUFAs compared with the placebo for hyperactive behaviours (including disobedience, distractibility, and impulsivity) and stereotypy, each with a large effect size. Statistics indicated a trend toward superiority of n-3 fatty acids over the placebo for hyperactivity [110]. 

The second study with positive findings was a 16-week trial performed by Yui and colleagues [111]. They evaluated the efficacy of supplementation with large doses of DHA and AA (240 mg/d of DHA and 240 mg/d of AA) or placebo in 13 participants (6–28 years old, mean age 14.6 years). Supplementation regimen significantly improved behavioural aberration such as social withdrawal. Treatment effect sizes were more favourable for the treatment group compared with the placebo group.

An open-label trial with a larger sample was performed by Ooi and colleagues [112] that examined the efficacy of a 12-week n-3 PUFA supplementation among 41 children and adolescents aged 7–18 years and diagnosed with ASD. At post-treatment, blood fatty acid levels were significantly correlated with changes in the core symptoms of ASD and baseline levels of blood fatty acids were also predictive of response to the n-3 PUFA treatment. Participants showed significant improvements in the Social and Attention Problems Syndrome Scales of the Child Behaviour Checklist and in all subscales of the Social Responsiveness Scale.

In more recent years, Keim and colleagues conducted a placebo controlled trial to prove the effectiveness of PUFA supplementation on ASD symptoms, but significant effects were confined to only one assessment scale [113]. In this 90 day randomised, fully blinded, placebo-controlled trial, the authors enrolled 31 children aged 18–38 months who were born at ≤29 weeks of gestation. One group was assigned to receive PUFA treatment (including 338 mg EPA, 225 mg DHA, and 83 mg GLA per day), while the other group received canola oil (124 mg palmitic acid, 39 mg stearic acid, 513 mg linoleic acid, 225 mg α-linolenic acid, and 1346 mg oleic acid). They found clinically significant improvements in ASD symptoms for children assigned to receive PUFA treatment measured by the Brief Infant Toddler Social and Emotional Assessment (BITSEA) ASD Scale, but no significant effects were observed on other outcome measures.

Discordant and discouraging findings were reported by four other placebo-controlled studies [114,115,116,117]. The study performed by Johnson and colleagues [114] was a prospective, open label, parallel group trial on n-3 PUFA supplement (*n* = 10) compared to a healthy, low sugar diet (*n* = 13) for children with ASD. Participants in the PUFA group took a daily dose of 400 mg of DHA for three months. No clinical improvements were observed on any of the behavioural or developmental outcome measures. Similarly, Bent and collaborators examined 27 children aged three to eight years with ASD who received 1.3 g/day of n-3 PUFAs (350 mg of EPA and 230 mg of DHA) for 12 weeks. Even if there was a reduction in hyperactivity in the treatment versus the placebo group, it was not statistically significant (*p* = 0.40 and *p* = 0.83, respectively) [115]. Unfavourable results were also obtained by Voigt and colleagues [116], who examined the clinical answer of a six month dietary supplementation of n-3 PUFAs in 48 children 3–10 years of age with ASD randomised to receive 200 mg/day of DHA (*n* = 24) or the placebo (*n* = 24). The authors did not show any improvements in core symptoms of autism.

Parellada and colleagues [117] investigated the effect of 8-weeks of supplementation with n-3 PUFAs (962 mg/d and 1155 mg/d for children and adolescents, respectively) in 68 children and adolescents with ASD. Treatment with n-3 PUFAs improved the erythrocyte membrane ω6/ω3 ratio in comparison to the placebo group. However, the authors did not find a significant difference in behavioural measures (Social Motivation and Social Communication subscales score) between groups. In summary, there is a dearth of scientific evidence to support the effectiveness of omega-3 fatty acids for ASD, with discordant findings among available investigations. Larger randomised controlled trials with appropriate dosage and duration of PUFA supplementation are needed to sort out this issue [118].

Results of the RCTs are displayed in Table 6.

### 4.8. Eating Disorders

Eating disorders (ED) are complex diseases that impact on both the physical and socio-emotional health of young people [119]. Research carried out worldwide indicates that a high proportion of adolescents encounter eating problems [120,121,122,123]. Among the spectrum of abnormal eating attitudes and concerns, anorexia nervosa (AN) represents the most severe disorder. Three studies with female patients revealed that essential fatty acid status in plasma phospholipids and erythrocyte membranes is altered in eating disorders with weight loss such as anorexia nervosa [124,125,126]. One trial [125] the investigated PUFA levels of 17 patients (mean age was 16.8 ± 2.3 years) hospitalised for anorexia nervosa, compared with 11 healthy females. Patients suffering from anorexia nervosa showed a lower n-6 PUFA level in plasma phospholipids, but higher DHA compared to the controls. Ten years later, Holman et al. [124] studied eight patients with anorexia nervosa and 19 controls (<25 years old) and identified elevated content of γ-linolenic acid, normal levels of α-linolenic acid, but a decrease in the other PUFAs in the phospholipid profile. The authors hypothesised that patients with anorexia nervosa have deficiencies of selected essential fatty acids. These data were not confirmed by the study performed by Zak and colleagues [126], which enrolled 16 young women suffering from AN (mean age 22.5 years) and 25 healthy control women (mean age 22.4 years). A decreased level of n-6 PUFA concentration was reported in line with the study by Langan and Farrel, but the authors did not find significant differences in the n-3 PUFA concentrations.

Three other investigations more specifically focused on adolescents with ED (anorexia nervosa, bulimia nervosa, and ED not otherwise specified) [127,128,129] found that the proportions of n-3 PUFAs in phospholipids and erythrocyte membranes did not differ between the patients and controls. However, a subset of patients with comorbid depression exhibited lower proportions of DHA compared to those without depression. In particular, Swenne and colleagues [128] examined the relationship between weight changes and essential fatty acids in plasma phospholipids and erythrocyte membranes of 220 adolescent girls with eating disorders (ED). Participants had a mean age of 15.3 years. Results showed that the proportions of EPA and DHA did not differ from the controls, but the composition of phospholipids and erythrocyte membranes differed between patients with ED and controls for the majority of the other fatty acids. In conclusion, according to Swenne’s results, the proportion of most fatty acids is influenced by weight changes, but the proportions of n-3 PUFA end products remain normal. When subjects were stratified by the presence and absence of comorbid depression (*n* = 84 and 133), the reduction of n-3 PUFA concentration was related to depression and was not influenced by differences in weight and duration of the disease [129].

The same authors also examined changes in the erythrocyte membrane’s fatty acid composition in 24 patients after 1-year of follow-up. Alterations of essential fatty acid status observed at the baseline largely normalised during treatment: PUFA status improved with weight gain. Nevertheless, the authors sustained that the normalisation of essential fatty acid status is attributed to adequate nutrition, weight gain, and the consequent return to normalisation of metabolism and endocrine function. Therefore, supplementation with n-3 PUFAs does not appear necessary in ED patients, because restoration of healthy eating behaviour and weight may lead to normalisation of PUFA markers [127].

Two studies evaluated the efficacy of n-3 PUFA supplementation as additional treatment for eating disorders in young children and adolescents [130,131]. The authors published a case report of a 15-year old refractory patient with severe anorexia nervosa [130]. The patient, after several months of traditional treatment, showed a rapid and long-lasting improvement by the end of three months of 1 g/day EPA supplementation. A significant improvement in both weight and food intake was reported. In a later study [131], they tested a small sample of seven patients diagnosed with anorexia nervosa aged between 13 and 22 years and treated for three months with EPA 1 g per day. They reported a significant improvement in mood after the first 6–8 weeks of treatment and this change was associated with better general functioning. In patients who stopped the EPA treatment, there was a deterioration in mood, and in weight and growth at about 2–3 months after the cessation of the treatment.

In conclusion, the findings of the clinical studies conducted in ED samples are largely insufficient to draw conclusions.

Results of the RCTs are displayed in Table 7.

### 4.9. Borderline Personality Disorder

The effects of PUFA supplementation have been studied almost exclusively in adult patients with personality disorders. To our knowledge, only one RCT has been conducted in a sample of adolescent patients [132]. The study was a post-hoc subgroup analysis of subjects with BPD who attended a treatment trial of n3-PUFAs in young people at UHR for psychosis (see previous paragraph on schizophrenia) [132]. The main trial enrolled 81 people at UHR and 15 of them (18.5%) received a consensus diagnosis of BPD (14 females, mean age 16.2 years). Participants were randomly assigned to two groups: eight subjects were treated with 1.2 g/day n-3 PUFAs (700 mg of EPA, 480 mg of DHA) and seven were treated with placebo. Both groups had comparable baseline characteristics and all patients either completed the 12-week intervention or made a transition to psychosis during this period. In the group who received n-3 PUFAs, the conversion rates to psychotic disorder at 12 weeks were 0.0% (0/8) while it was 28.6% (2/7) in the placebo group. The n-3 PUFAs group also showed a significant improvement in functioning (measured by the Global Assessment of Functioning, GAF) and a significantly greater response on symptoms (assessed by the Positive and Negative Syndrome Scale, PANSS) compared with those in the placebo group. Findings suggest that PUFAs may be an effective and well-tolerated treatment in adolescents with BPD who also meet UHR criteria for psychotic symptoms. Notably, the magnitudes of group differences ranged from large (negative, general, total, and BPD symptoms) to very large (Global Assessment of Functioning). The correlation of n-3 PUFA levels with psychopathology and functioning further support the efficacy of PUFAs in these patients and it takes on even more significance because functioning is one of the most important outcomes for clinical trials in BPD patients [133]. 

## 5. Conclusions

On the basis of the studies previously reported and discussed, the following considerations can be made. In line with trials conducted in samples of adult patients, the main evidence of efficacy for n-3 PUFA supplementation has been obtained in mood disorders. In particular, supplementation with a daily dose ranging between 0.6 g and 2.5 g of EPA and DHA was found to be effective in reducing depressive symptoms in major depressive disorders, bipolar disorder, and anorexia nervosa. Rather recent findings [134] suggest a potential beneficial role of omega-3 fatty acids in addition to stable medications in treating mainly depressive symptoms, but also manic symptoms at the approximate dose of 1–2 g/day in children and adolescents with bipolar disorder. We can also deduce from follow-up studies [68] that long-lasting treatments lead to a more stable and persistent improvement in depressive symptoms.

With regard to schizophrenia, the target population of trials considered in this review was that of children and adolescents at high risk of developing psychosis. From the data of available studies, it is possible to infer that supplementation with a daily dose of 1–2 g of n3-PUFA has a protective effect on the conversion rate in psychosis and can also produce positive effects in the early phases of schizophrenia (first episode of psychosis) [50,135]. 

The effect of n3-PUFA supplementation in anxiety disorders cannot be established due to the scarcity of data, but available results are encouraging: two of four studies conducted in non-psychiatric populations showed a significant reduction in anxiety symptoms and one of two trials carried out on subjects with anxiety disorders reported that n-3 PUFAs were useful in treating symptoms of depression and anxiety. Results regarding obsessive compulsive disorder and borderline personality disorder in children and adolescents are insufficient to draw any conclusions on the efficacy of PUFAs in these periods of life. However, the promising results obtained in adult patients with BPD treated with the supplementation of EPA and DHA [27,136,137,138] suggest the opportunity to further test these agents in the treatment of young patients. In ADHD, a few promising results have been obtained using the association of omega-3 and omega-6 fatty acids. Several trials comparing the combination of these agents with commonly used medications found an equivalent improvement of ADHD related symptoms. Larger trials are now required to replicate these findings, and to establish the duration of treatment effects as well as the optimal formulations and doses. Available evidence in autism spectrum disorders is very weak. Most favourable findings concern the improvement in social interactions and control of hyperactive behaviours with a combination of high dose EPA and DHA. In addition, in studies of young patients with eating disorders, the findings were inconsistent and discordant.

The majority of RCTs reported that omega-3 fatty acids were well tolerated. The lack of significant adverse effects is a reason to consider the potential role of these agents, especially in the treatment of young individuals. PUFA supplementation can be considered as a promising and self-therapeutic option in children and adolescents, but further investigations are required to make clear which are the more specific clinical targets, to define the better modalities of administration (doses, duration of treatment, PUFAs compounds and combination), and to provide reliable guidelines for the use of these agents in clinical practice.

## Figures and Tables

**Table 1:** Schizophrenia and BPD.

	Study Design	Country	No.	Age Group	Disease	Daily Dosage (g/d)	Duration	Rating Scale	Outcome
Amminger et al., 2010 [50]	Randomised, double-blind, placebo controlled trial	Austria	81	13–25 years	- Individuals at ultra-high risk of psychotic disorder.	1.2g/d n-3 PUFAs or placebo	12 weeks	- PANSS - MADRS - GAF - SCID-I/P	The cumulative conversion rates to psychotic disorder were 4.9% (2 of 41) in the n-3 group and 27.5% (11 of 40) in the placebo group.
Amminger et al., 2015 [53]	Post hoc subgroup analysis	Austria	81	13–25 years	- Individuals at ultra-high risk of psychotic disorder. - BPD	1.2g/d n-3 PUFAs or placebo	12 weeks	- PANSS - MADRS - GAF	N-3 PUFAs provided improvements in functioning and symptoms.
Mcgorry et al., 2017	Randomised, double-blind, placebo controlled trial	Australia	304	13–40 years	- Individuals at ultra-high risk of psychotic disorder.	1.4 g/d n-3 PUFAs together with CBCM or placebo with CBCM or placebo	24 weeks	- YMRS - BPRS - CDRS	No significant difference between ω-3 PUFAs and placebo in transition rate.
Pawełczyk et al., 2016 [51]	Randomised, double-blind, placebo controlled trial	Poland	71	16–35 years	-Individuals at first-episode schizophrenia	2.2 g/d n-3 PUFAs or placebo	26 weeks	- PANSS - CDSS - GAF - CGI-S	50% improvement in total PANSS score was achieved significantly more frequently in the n-3 PUFAS group than in the placebo group.

BPD: Borderline Personality Disorder; BPRS: Brief Psychosis Rating Scale; CDRS: Children’s Depression Rating Scale; CDSS: Calgary Depression Scale for Schizophrenia; CGI-S Clinical Global Impressions scale; GAF: Global Assessment of functioning MADRS: Montgomery-Åsberg Depression Rating Scale; PANSS: Positive and Negative Syndrome Scale; SCID-I/P: The Structured Clinical Interview for DSM-IV Axis I Disorders; YMRS: Young Mania Rating Scale.

**Table 2:** Bipolar disorders.

	Study Design	Country	No.	Age Group	Disease	Daily Dosage (g/d)	Duration	Rating Scale	End Point
Wozniak et al., 2007 [59]	Open label trial	USA	20	6–17 years	- Bipolar spectrum disorder	1290 mg–4300 mg/d n-3 PUFAs	8 weeks	- YMRS - BPRS - SANS - MADRS	8.9 ± 2.9 point reduction in the YMRS scores (*p* < 0.001).
Clayton et al., 2009 [61]	Open label trial	Australia	18	mean age = 16.1 ± 0.81 years	- Bipolar spectrum disorder	360 mg/day EPA and 1560 mg/day DHA	6 weeks	- YMRS - HAM-D - C-GAS) - CBCL-PR,	Clinician ratings of YMRS and HAM-D were significantly lower (*p* = 0.004 and *p* = 0.002) and C-GAS significantly higher (*p* < 0.001) following supplementation.
Fristad et al., 2015 [64]	Randomised controlled trial	USA	23	7–14 years	- BP-NOS - CYC	2000 mg/day of n-3 PUFAs versus placebo and IF-PEP versus AM using a 2 · 2 design (O3 + PEP: *n* = 5; O3 + AM: *n* = 5; placebo + PEP: *n* = 7; placebo + AM: *n* = 6)	12 weeks	- K-SADS - KDRS - KMRS - CDRS-R - YMRS	Manic symptoms improved over time without significant treatment effects. Effect of IF-PEP on child depression compared with AM was medium (d = 0.63, CDRS-R) to large (d = 1.24, KDRS). Effect of n-3 PUFAs on depression was medium (d = 0.48, KDRS).
Vesco et al., 2018 [68]	Randomised controlled trial	USA	95	7–14 years	- BP-NOS or CYC (*n* = 23) - MDD (*n*= 72)	- 1.87 g/d n3-PUFAs in monotherapy - PEP monotherapy - PEP + n3-PUFAs	12 week	- BRIEF - GEC - BRI - YMRS	PUFAs supplementation were associated with significant improvements in executive functions and in dysphoric mood, irritability, and self-esteem.
Fristad et al., 2021 [67]	Naturalistic follow-up study	USA	38	11–19 years	- BP-NOS or CYC (*n* = 13) - MDD (*n* = 25)		2–5 years after participation in randomised clinical trials (RCTs) Fristad et al., 2015	- Mental Health Services and Medication Grids - CDRS - YMRS - BRIEF - CGAS - The OATS Family Experience Assessment—Child and Parent Report (FEA)	Compared to baseline depressive symptoms, participants had significantly lower CDRS-R scores at follow-up, with a small effect size and were functioning better (CGAS scores) with a medium effect size. Manic symptom severity, executive functioning, and global functioning remained comparable to end of RCT. The majority of parents and youth reported improved youth emotion regulation skills and family communication.

BPRS: Brief Psychosis Rating Scale; BRI: Behaviour Regulation; BRIEF: Behaviour Rating Inventory of Executive Functioning; CBCL-PR: Child Behaviour Checklist—Parent Report; CDRS: Children’s Depression Rating Scale; CDSS: Calgary Depression Scale for Schizophrenia; C-GAS: Global Assessment Scale for Children; CGI-S Clinical Global Impressions scale; GAF: Global Assessment of functioning; GEC: Global Executive Composite; HAM-D: Hamilton Depression Rating Scale; K-SADS: Kiddie Schedule for Affective Disorders ( KRDS Depression; KMRS: mania); MADRS: Montgomery–Åsberg Depression Rating Scale; PANSS: Positive and Negative Syndrome Scale; SANS: Schedule for Assessment of Negative Symptoms; SCID-I/P: The Structured Clinical Interview for DSM-IV Axis I Disorders; YMRS: Young Mania Rating Scale.

**Table 3:** Depressive disorder.

	Study Design	Country	No.	Age Group	Disease	Daily Dosage (g/d)	Duration	Rating Scale	End Point
Nemets et al., 2006 [76]	Randomised controlled trial	Israel	28	6–12 years	- MDD	400 mg/d EPA and 200 mg/d DHA or placebo	4 weeks	- CDRS - CDI - CGI	Highly significant effects of omega-3 on symptoms using the CDRS (*p* = 0.003), CDI (*p* < 0.005), and CGI (*p* = 0.002).
Young et al., 2017 [78]	Randomised controlled trial	USA	72	7–14 years	- MDD (*n* = 37) - dysthymic disorder (*n* = 5) -depressive disorder not otherwise specified (*n* = 30)	- 2 g/d n3-PUFAs in monotherapy - PEP in monotherapy- PEP + n3-PUFA - placebo	12 weeks	- SNAP-IV - ECBI	N-3 PUFAs yielded more favourable trajectories than placebo on the SNAP-IV Hyperactivity/Impulsivity subscale (*p* = 0.034, d = 0.44) and marginally more favourable on Total (*p* = 0.080, d = 0.42), and Inattention scores (*p* = 0.059, d = 0.49).
Vesco et al., 2018 [68]	Randomised controlled trial	USA	95	7–14 years	- BP-NOS or CYC (*n* = 23) - Depressive disorder (*n* = 72)	- 1.87 g/d n3-PUFAs in monotherapy - PEP monotherapy - PEP + n3-PUFAs	12 weeks	- BRIEF - GEC - BRI - YMRS	PUFAs supplementation were associated with significant improvement in executive functions and in dysphoric mood, irritability, and self-esteem.
Trebatická et al., 2020 [77]	Randomised, double-blind, placebo controlled trial	Slovakia	60	7–18 years	-MDD (*n* = 31) -Mixed anxiety and depressive disorder (*n* = 29)	2400 mg of total omega-3 PUFAs/day or placebo	12 weeks	- CDI	PUFAs supplementation were associated with reductions in Children’s Depression Inventory (CDI) scores.

BRI: Behaviour Regulation; BRIEF: Behaviour Rating Inventory of Executive Functioning; CDI: Child Development Inventory; CDRS: Children’s Depression Rating Scale; CGI Clinical Global Impressions; GEC: Global Executive Composite; ECBI: Eyberg Child Behaviour Inventory; SNAP-IV: Swanson, Nolan, and Pelham-IV.

**Table 4:** Anxiety disorder.

	Study Design	Country	No.	Age Group	Disease	Daily Dosage (g/d)	Duration	Rating Scale	End Point
Hamazaki et al., 2005 [83]	Randomised, double-blind, placebo controlled trial	Japan	22	18–24 years	Healthy subjects	- EPA 400 mg/d + DHA 300 mg/d Or placebo	8 weeks	EP, NE, dopamine, cortisol and corticotropin concentration	PUFAs supplementation were associated with a decreased activation of the hypothalamic–pituitary–adrenal (HPA) axis.
Kiecolt-Glaser et al., 2011 [84]	Randomised, double-blind, placebo controlled trial	USA	68	21–29 years	Healthy subjects	EPA 2085 mg/d + DHA 348 mg/d or placebo	12 weeks	-Beck Anxiety Inventory	PUFAs supplementation were associated with 20% reduction in anxiety symptoms, without significant change in depressive symptoms.
Manos et al., 2018 [85]	Randomised, double-blind, placebo controlled trial	USA	24	12–21 years	- anxiety disorder and AN	- EPA 2120 mg/d + DHA 600 mg/d or placebo	12 weeks	- BAIT - CES-D - EAT-26	No evidence that omega-3 PUFA benefited anxiety.
Robinson et al., 2019 [86]	Randomised, double-blind, placebo controlled trial	USA	50	15–40 years	- early psychosis and anxiety	- EPA 740 mg/d and DHA 400 mg/d or placebo	16 weeks	- BPRS - SANS - CGI - SAFTEE-SI	Implementation with n-3 PUFAs was found mainly useful on depression-anxiety domain measured with the Brief Psychiatric Rating Scale (BPRS).

BAIT: Beck Anxiety Inventory-Trait; BPRS: Brief Psychosis Rating Scale; CES-D: Centre for Epidemiologic Studies Depression Scale; CGI Clinical Global Impressions; EAT-26: Eating Attitudes Test; SAFTEE-SI: Systematic Assessment for Treatment Emergent Events Specific Inquiry; SANS: Schedule for Assessment of Negative Symptoms.

**Table 5:** ADHD.

	Study Design	Country	No.	Age Group	Disease	Daily Dosage (g/d)	Duration	Rating Scale	End Point
Voigt et al., 2001 [98]	Randomized, double-blind, placebo controlled trial	USA	63	6–12 years	ADHD	- DHA 345 mg/d or placebo	16 weeks	-TOVA - Children’s Colour Trails test - CBCL - CRS	Supplementation does not decrease symptoms of ADHD.
Richardson & Puri, 2002 [99]	Randomized, double-blind, placebo controlled trial	UK	41	8–12 years	Learning disabilities	EPA186 mg/d + DHA 480 mg/d + GLA 96 mg/d + vitamin E 60 IU/d + AA 42 mg/d + LA (864 mg/d +thyme oil 8 mg/d or placebo	12 weeks	CPRS-L	Significant benefit in alleviating many ADHD-related symptoms in children with specific learning difficulties.
Harding et al., 2003 [102]	double-blind, placebo controlled trial	USA	20	7–12 years	ADHD	EPA 180 mg/d + DHA 120 mg/d + GLA 45 mg/d OR methilphenidate	4 weeks	- IVA/CPT	Supplementation resulted in equivalent improvements in attention and self-control as methylphenidate.
Hirayama et al., 2004 [105]	Randomized, double-blind, placebo controlled trial	Japan	40	6–12 years	ADHD	DHA 515 mg/d + EPA 100 mg/d or placebo	8 weeks	- AD/HD-related symptoms - Aggression assessment- Development Test of Visual Perception - Visual and auditory short-term memory; - Developmental test of visual–motor integration – Continuous performance test - Impatience test.	No improvement of ADHD.
Widenhorn-Müller et al., 2014 [101]	Randomized, double-blind, placebo controlled trial	Germany	95	6–12 years	ADHD	EPA 600 mg/d +DHA 120 mg/d or placebo	16 weeks	- CBCL - TRS	No effect on cognitive measures and parent- and teacher-rated behaviour.
Wu et al., 2015 [104]	Randomized, double-blind, placebo controlled trial	China	179	7–12 years	ADHD (*n* = 91) Lower IQs (*n* = 88)	eggs rich in n-3 PUFAs or normal eggs	12 weeks	- Mann–Whitney test - Wilcoxon Signed Ranks test	Improvements both in visual acuity and in the RBC fatty acid profile.

CBCL: Child Behaviour Checklist; CPRS-L: Conners’ Parent Rating Scale; CRS: Conners Rating Scales; IVA/CPT: Intermediate Visual and Auditory/Continuous Performance Test; TRS: Teacher’s Report Form; TOVA: Test of Variables of Attention.

**Table 6:** ASD.

	Study Design	Country	No.	Age Group	Disease	Daily Dosage (g/d)	Duration	Rating Scale	Outcome
Amminger et al., 2007 [110]	Randomised, double-blind, placebo controlled trial	Austria	13	5–17 years	ASD	EPA 840 mg/d + DHA 700 mg/d Or placebo	6 weeks	- ABC	Advantage of n-3 PUFAs compared with placebo for hyperactive behaviours and stereotypy.
Johnson et al., 2010 [114]	Prospective, open label trial	USA	23	44.7 m (s.d. = 7.63) years	ASD (*n* = 17)PDD, NOS (*n* = 6)	DHA 400 mg/d	12 weeks	- CBCL - ADOS	No clinical improvements were observed.
Bent et al., 2011 [115]	Pilot randomised placebo controlled trial	USA	27	3–8 years	ASD	EPA 350 mg/d + DHA 230 mg/d or placebo	12 weeks	- SRS - CGI - BASC - ABC	Not statistically significant improvements in the treatment group vs. the placebo.
Yui et al., 2012 [111]	Randomised, double-blind, placebo controlled trial	Japan	13	6–28 years	ASD	DHA 240 mg/d + ARA 240 mg/dor placebo	16 weeks	- SRS - ABC	Improvement of social deficit in individuals with ASD.
Voigt et al., 2014 [116]	Randomised, double-blind, placebo controlled trial	USA	48	3–10 years	ASD	DHA 200 mg/d or placebo	24 weeks	- CGI-I - ABC - CDI - BASC	No improvement in core symptoms of autism.
Ooi et al., 2015 [112]	Open label trial	Singapore	41	7–18 years	ASD	DHA 840 mg/d + EPA 192 mg/d	12 weeks	- SRS-P - CBCL	Significant improvements on all subscales of the Social Responsiveness Scale (*p* < 0.01) and the Social and Attention Problems syndrome scales of the Child Behaviour Checklist (*p* < 0.05).
Parellada et al., 2017 [117]	Randomized, crossover, placebo controlled trial	SPAIN	68	5–17 years	ASD	PUFAs 962 mg/d for children and 1155 mg/d for adolescents or placebo	8 weeks	- SRS	No treatment effect.
Keim et al., 2018 [113]	Randomized, double-blind, placebo controlled trial	USA	31	18–38 years	ASD	EPA 338 mg/d + DHA 225 mg/d + GLA 83 mg/d or placebo	12 weeks	- BITSEA - PDDST-II - Ages and Stages Questionnaire-Social Emotional	Significant improvements in ASD symptoms measured by the BITSEA, but no significant effects were observed on other outcome measures.

ABC: Aberrant Behaviour Checklist; ADOS: Autism Diagnostic Observation Schedule; ASD: Autism Spectrum Disorder; BASC: Behaviour Assessment Scale for Children; BITSEA: Brief Infant Toddler Social and Emotional Assessment; CBCL: Child Behaviour Checklist; CGI: Clinical Global Impressions; PDDST-II: Pervasive Developmental Disorders Screening Test II; SRS (-P): Social Responsiveness Scale (–Parent).

**Table 7:** Eating disorders.

	Study Design	Country	No.	Age Group	Disease	Daily Dosage (g/d)	Duration	Rating Scale	Outcome
Ayton et al., 2004 [130]	Case report	UK	1	15 y	AN	EPA 1 g/day	12 weeks	BMI	Improvements in both weight and food intake.
Ayton et al., 2004 [131]	Pilot open case series	UK	10	13–22 y	AN	EPA 1 g/day	6–8 weeks	BMI	Improvement of general functioning and mood.

BMI: Body mass index.

## Data Availability

Not applicable.

## Abbreviations

ABC	Aberrant Behaviour Checklist
AM	Active monitoring
AN	Anorexia nervosa
AA	Arachidonic Acid
ADHD	Attention deficit hyperactivity disorder
ADOS	Autism Diagnostic Observation Schedule
ASD	Autism Spectrum Disorder
BAIT	Beck Anxiety Inventory-Trait
BASC	Behaviour Assessment Scale for Children
BRIEF	Behaviour Rating Inventory of Executive Functioning
BRI	Behaviour Regulation
BP-NOS	Bipolar disorder not otherwise specified
BPD	Borderline Personality Disorder
BITSEA	Brief Infant Toddler Social and Emotional Assessment
BPRS	Brief Psychosis Rating Scale
CDSS	Calgary Depression Scale for Schizophrenia
CES-D	Centre for Epidemiologic Studies Depression Scale
CBCL	Child Behaviour Checklist
CBCL-PR	Child Behaviour Checklist—Parent Report
CDI	Child Development Inventory
CDRS	Children’s Depression Rating Scale
CGI-S	Clinical Global Impressions scale was used to measure symptom severity
CHR	Clinical High Risk
CBCM	Cognitive behavioural case management
CPRS-L	Conners’ Parent Rating Scale
CYC	Cyclothymic disorder
DHA	Docosahexaenoic acid
EAT-26	Eating Attitudes Test
EA	Eating Disorder
EPA	Eicosapentaenoic acid
EFAs	Essential Fatty Acids
ECBI	Eyberg Child Behaviour Inventory
GAF	Global Assessment of functioning
C-GAS	Global Assessment Scale for Children
GEC	Global Executive Composite
HAM-D	Hamilton Depression Rating Scale
IF-PEP	Individual family
IVA/CPT	Intermediate Visual and Auditory/Continuous Performance Test
K-SADS	Kiddie Schedule for Affective Disorders Rating Scales
KRDS	Depression
KMRS	Mania
LA	Linolenic acid
MDD	Major depressive disorder
MADRS	Montgomery-Åsberg Depression Rating Scale
n-3 PUFAs	Omega 3 Polyunsaturated Fatty Acids
n-6 PUFAs	Omega 3 Polyunsaturated Fatty Acids
PDD NOS	Pervasive Developmental Disorder, Not Otherwise Specified
PDDST-II	Pervasive Developmental Disorders Screening Test II
PANSS	Positive and Negative Syndrome Scale
PEP	psychoeducational psychotherapy
SANS	Schedule for Assessment of Negative Symptoms
SDQ	Strengths and Difficulties Questionnaire
SNAP-IV	Swanson, Nolan, and Pelham-IV
SAFTEE-SI	Systematic Assessment for Treatment Emergent Events Specific Inquiry
TRS	Teacher’s Report Form
TOVA	Test of Variables of Attention
SRS-P	The Social Responsiveness Scale–Parent
UHR	Ultra-High-Risk
YMRS	Young Mania Rating Scale
GLA	γ-linolenic acid
